# FGF21 and SHBG as Putative Hepatic Axes in Maternal Metabolic Adaptation: A Hypothetical Framework for Postpartum Insulin Sensitivity Restoration

**DOI:** 10.3390/biom16070998

**Published:** 2026-07-08

**Authors:** Kornelia Purc-Bandurko, Katarzyna Trojnar, Angelika Masiarz, Adrian Bandurko, Żaneta Kimber-Trojnar, Bożena Leszczyńska-Gorzelak

**Affiliations:** 1 Chair and Department of Obstetrics and Perinatology, Medical University of Lublin, 20-090 Lublin, Poland; bozena.leszczynska-gorzelak@umlub.edu.pl; 2 Student’s Scientific Association at the Chair and Department of Obstetrics and Perinatology, Medical University of Lublin, 20-090 Lublin, Poland; katarzynaatrojnar@gmail.com (K.T.); masiarz.ang@gmail.com (A.M.); 3 Department of Urology, Center of Oncology of the Lublin Region, 20-090 Lublin, Poland; adrian.bandurko@gmail.com

**Keywords:** pregnancy, insulin resistance, hepatokines, FGF21, SHBG, gestational diabetes mellitus, postpartum metabolic adaptation, metabolic flexibility, type 2 diabetes

## Abstract

Pregnancy is a physiological state of transient, reversible insulin resistance accompanied by major adaptations in glucose and lipid metabolism. Although placental hormones are key drivers of gestational insulin resistance, the mechanisms underlying the rapid restoration of insulin sensitivity after delivery remain incompletely understood. This review proposes a conceptual framework in which fibroblast growth factor 21 (FGF21) and sex hormone-binding globulin (SHBG) are considered as complementary hepatic signals potentially involved in maternal metabolic adaptation. During late pregnancy, FGF21 may function as a metabolic stress-response factor associated with fatty acid oxidation, lipid handling, and mitochondrial adaptation through AMPK–PPARα-related pathways. Reduced SHBG, in contrast, may reflect hepatic insulin resistance and altered hepatic metabolic regulation. After delivery, changes in FGF21 and SHBG levels may be associated with recovery of hepatic metabolic homeostasis and insulin sensitivity, while persistent adaptive FGF21 signaling facilitate metabolic reprogramming may contribute to ongoing metabolic adaptation. Postpartum metabolic recovery may therefore represent an active and dynamic process rather than a purely passive consequence of placental hormone withdrawal. Disruption of FGF21- and SHBG-mediated pathways may contribute to persistent insulin resistance and increased cardiometabolic risk after gestational diabetes. Understanding hepatokine-mediated regulation of maternal metabolic flexibility may provide further insight into postpartum metabolic recovery and may support future development of risk stratification strategies, biomarker-based approaches, and preventive interventions aimed at reducing the risk of type 2 diabetes after pregnancy.

## 1. Introduction

### 1.1. Pregnancy as a Model of Reversible Insulin Resistance

Pregnancy is a unique physiological model of transient and reversible insulin resistance associated with maternal metabolic remodeling, in which insulin sensitivity decreases by more than 50% during the second and third trimesters. This phenomenon represents an evolutionarily programmed strategy for the redistribution of energy substrates to the fetoplacental unit. Placental hormonal activity, particularly placental growth hormone (PGH) and human placental lactogen (hPL), leads to a systemic reprogramming of maternal metabolism.

Glucose-stimulated insulin secretion and pancreatic β-cell mass are increased by hPL and prolactin [1,2,3,4], thereby supporting compensatory hyperinsulinemia during normal pregnancy.

In late pregnancy, the placenta releases pro-inflammatory cytokines, including tumor necrosis factor alpha (TNF-α), which modulate insulin receptor substrate-1 (IRS-1) phosphorylation, thereby enhancing peripheral insulin resistance [5,6]. This process reduces glucose uptake by maternal tissues and ensures its availability to the fetus.

Importantly, this insulin-resistant state is rapidly reversed after delivery [7], highlighting the dynamic and reversible nature of maternal metabolic adaptation.

### 1.2. Differences Between Physiological and Pathological Insulin Resistance

While this dynamic reversibility defines normal gestation, the boundary between physiological adaptation and metabolic failure can become compromised.

The key distinction between normal pregnancy and gestational diabetes mellitus (GDM) lies in the compensatory capacity of pancreatic β-cells [8]. Whereas normal pregnancy is characterized by adaptive hyperinsulinemia that maintains glucose homeostasis, GDM develops when β-cell compensation becomes insufficient in the presence of increasing metabolic demands and insulin resistance [6,9,10].

Physiological insulin resistance enhances glucose availability for the fetus, utilizing resources accumulated within the maternal body since early pregnancy. To maintain euglycemia, the liver upregulates gluconeogenesis and modulates lipid metabolism. Concurrently, gestational hormones stimulate de novo lipogenesis, resulting in a marked elevation in plasma triglyceride concentrations, predominantly during the third trimester. Furthermore, hepatic amino acid metabolism undergoes remodeling, characterized by the inhibition of ureagenesis to conserve nitrogen essential for fetal growth. Additionally, the liver secretes specific hepatokines that regulate systemic insulin sensitivity and placental nutrient transport.

In GDM, hepatic insulin resistance is pathologically exacerbated, driving uncontrolled gluconeogenesis, maternal hyperglycemia, and an increased risk of fetal macrosomia. Concomitant derangements in lipid metabolism contribute to oxidative stress, hepatic steatosis, and elevated liver enzyme activity. Maternal obesity further aggravates this intrahepatic pro-inflammatory environment, thereby impairing insulin signaling. Finally, dysfunctional bile acid receptor signaling and aberrant hepatokine secretion exacerbate metabolic dysregulation, ultimately compromising fetal development and the long-term health outcomes of the offspring [11,12,13,14,15].

### 1.3. Research Gap

A defining feature of pregnancy-associated insulin resistance is its rapid reversal after delivery, often occurring within days postpartum. Defining the mechanisms underlying this process is crucial, as impaired postpartum metabolic adaptation may contribute to the progression of type 2 diabetes mellitus (T2DM) [16].

However, the molecular mechanisms governing this postpartum “metabolic reset” remain incompletely understood.

Emerging evidence suggests that hepatokines, which integrate hormonal, lipid, and inflammatory signals between the liver and peripheral tissues, may play an important role in this process [3,17,18,19].

### 1.4. Two Hepatokine Axes

During pregnancy, the liver functions as an endocrine organ, integrating metabolic, inflammatory, and hormonal signals [18].

Among hepatokines, fibroblast growth factor 21 (FGF21) and sex-hormone-binding globulin (SHBG) are of particular importance, reflecting different aspects of metabolic adaptation and hepatic insulin sensitivity.

We propose that maternal metabolic adaptation during pregnancy and postpartum restoration of insulin sensitivity are coordinated by two complementary axes:

- The FGF21 axis, responsible for adaptation to metabolic stress and lipid overload;
- The hepatocyte nuclear factor 4 alpha (HNF4α)–SHBG axis, reflecting restoration of hepatocellular function after delivery.

The model has several important limitations. The proposed coordinated FGF21—SHBG signaling pathway remains a purely conceptual framework. Importantly, both factors are also synthesized by the placenta, which confounds the assessment of their direct hepatic impact on the maternal organism. While FGF21 and SHBG are readily measurable, circulating biomarkers, their master transcriptional regulator-HNF4alpha is an intracellular nuclear factor. Consequently, it cannot be directly quantified via routine peripheral blood analysis. The model assumes an adaptive, protective nature of the FGF21 axis in response to lipid overload. However, in pathological states such as overt gestational diabetes or obesity, target cells lose sensitivity to FGF21 due to a drastic downregulation of the B-Klotho co-receptor expression.

Due to the ethical and practical constraints of investigating the mechanisms of insulin resistance at a deep molecular level in humans, genetically engineered knock-out models, particularly murine models, were developed. In research focusing on insulin resistance and GDM, global insulin receptor knock-out models proved unviable, as mice lacking this receptor systematically succumbed to severe ketoacidosis within a few days of birth. Consequently, tissue-specific knock-out models represented a major breakthrough, enabling the selective ablation of the insulin receptor (IR) or its substrates (IRS-1, IRS-2) within a single organ. To date, attempts to investigate these relationships in mammals have relied on knock-out models—including LIRKO (Liver Insulin Receptor Knockout), BIRKO (Beta-cell Insulin Receptor Knockout), and selective IRS-1 and IRS-2 ablation—which accurately map isolated hepatic insulin resistance but fail to precisely recapitulate this specific dual axis during pregnancy. Therefore, future studies utilizing inducible, tissue-specific knock-out models are indispensable to definitively validate the postulated crosstalk between these metabolic axes during pregnancy [13,14,19,20].

Collectively, we hypothesize that postpartum “metabolic reset” may represent an actively regulated biological process involving coordinated changes in the FGF21 and HNF4α–SHBG axes. However, direct evidence demonstrating coordinated regulation or mechanistic crosstalk between these pathways remains limited, and the proposed framework should be regarded as hypothesis-generating.

## 2. Materials and Methods

A narrative literature review was conducted using the PubMed, Scopus, and Web of Science databases. Searches covered studies published up to April 2026 using combinations of the terms related to pregnancy, postpartum, insulin resistance, GDM, maternal insulin sensitivity, metabolic adaptation, FGF21, SHBG, HNF4α, hepatokines, and T2DM.

Preference was given to peer-reviewed articles published in English, including meta-analyses, systematic reviews, clinical studies involving human participants, prospective cohort studies, and mechanistic experimental studies relevant to maternal metabolic adaptation. Recent publications reflecting current scientific knowledge were prioritized, while selected seminal studies were retained when they provided fundamental physiological concepts essential for understanding the topic.

Case reports, conference abstracts, duplicate publications, and studies outside the scope of this review were excluded. The selected literature was evaluated qualitatively, and the findings were synthesized using a narrative approach.

As this study is based exclusively on previously published literature, no ethical approval was required.

## 3. Maternal Metabolic Adaptation and Postpartum Recovery

### 3.1. Metabolic Remodeling in Pregnancy: Glucose and Lipids

In early pregnancy, glucose and insulin levels remain close to pre-pregnancy values. In the second and third trimesters, insulin sensitivity decreases by approximately 50–60%, increasing nutrient availability for the fetus [21,22].

Maintenance of glucose homeostasis in late pregnancy requires increased hepatic gluconeogenesis, providing alanine as a key substrate. In GDM, impaired autophagy, accumulation of dysfunctional mitochondria, and endoplasmic reticulum stress are observed, suggesting compromised cellular metabolic flexibility [23,24].

Advanced pregnancy is characterized by hyperlipidemia with elevated triglycerides, very-low-density lipoprotein (VLDL), and low-density lipoprotein (LDL) fractions. This process is regulated by nuclear lipid receptors, including farnesoid X receptor (FXR). Rising estrogen levels increase VLDL synthesis, while lipoprotein lipase (LPL) activity in adipose tissue is reduced [19,25].

Pregnancy is associated with “accelerated starvation,” leading to enhanced ketogenesis during short-term fasting, resulting in increased circulating free fatty acids and ketone bodies after several hours of fasting [4,26,27].

In late pregnancy, enhanced lipolysis driven by placental hormones and increased non-esterified fatty acids (NEFA) shifts metabolism toward lipid oxidation. However, in GDM, this adaptive increase in lipid utilization is impaired, with reduced mitochondrial function and activation of pro-inflammatory fatty acid oxidation pathways in the placenta [12,28,29,30].

Hepatic insulin resistance and impaired gluconeogenesis regulation persist in GDM, accompanied by an abnormal adipokine profile and elevated triglycerides [31,32,33]. Collectively, these changes highlight the central role of the liver in integrating glucose and lipid metabolic adaptations during pregnancy and in their dysregulation in GDM.

### 3.2. Hormonal Regulation of Pregnancy-Induced Insulin Resistance

The main regulators of maternal metabolic adaptation are PGH and hPL. PGH is secreted continuously and impairs insulin signaling through modulation of IRS-1, thereby contributing to reduced maternal glucose uptake and increased substrate availability for the fetus [3,24], while hPL promotes lipolysis alongside cortisol-driven gluconeogenesis [24]. In parallel with these placenta-derived factors, ovarian steroids, including estrogens and progesterone, modulate glucose transporter type 4 (GLUT4) expression and insulin signaling in skeletal muscle, with progesterone additionally linked to oxidative stress and β-cell apoptosis in the pancreas [34,35,36].

### 3.3. Reversibility of Insulin Resistance After Delivery

Delivery leads to a rapid decline in diabetogenic hormones, including progesterone and hPL, initiating the rapid restoration of insulin sensitivity [37].

In women with GDM, abnormal IRS phosphorylation may maintain insulin resistance despite the disappearance of placental stimuli, suggesting partially persistent impairments in insulin signaling pathways.

In most patients, glycemia normalizes; however, some individuals exhibit residual β-cell dysfunction and low-grade chronic inflammation, increasing the risk of T2DM. Reduction in inflammatory cytokines improves glucose homeostasis and supports return to normoglycemia [3,38,39].

Postpartum metabolic recovery involves remodeling of hepatic and peripheral immune-metabolic functions [40], with gradual resolution of insulin-resistance-promoting signals and hepatocyte secretory remodeling. This transition reflects a coordinated shift from a pregnancy-adapted to a post-pregnancy metabolic state.

During pregnancy, liver volume increases by up to 20%, while postpartum involution occurs alongside remodeling of blood flow and amino acid metabolism [41]. Disturbances in this process may contribute to persistent metabolic dysfunction and increased risk of metabolic syndrome and non-alcoholic fatty liver disease (NAFLD) [19,20,27,42]. Together, these observations highlight the liver as a central organ in both the establishment and resolution of pregnancy-associated insulin resistance.

## 4. The Liver as a Central Integrator of Maternal Metabolic Plasticity

### 4.1. The Liver as an Endocrine Organ and Metabolic Hub

The liver is not only a metabolic organ but also an important endocrine regulator, integrating hormonal, immunological, and metabolic signals controlling systemic energy homeostasis. It participates in thyroid hormone metabolism, steroid metabolism, glucagon-like peptide-1 (GLP-1) signaling, hepatokine production, detoxification, and energy substrate redistribution [43,44].

It communicates with endocrine organs such as the pancreas, pituitary gland, thyroid, intestine, bone, and adrenal glands, while hormones themselves modulate hepatic synthetic and metabolic functions [18,20,43,45].

Due to its unique vascularization, the liver is constantly exposed to circulating and gut-derived signals, enabling rapid metabolic adaptation. Despite its relatively small mass (~2.5% of body weight), it receives up to 25% of cardiac output at rest [43,46]. This anatomical arrangement ensures continuous integration of nutrient, hormonal, and inflammatory signals.

Accordingly, hepatic dysfunction is closely associated with systemic insulin resistance and metabolic diseases such as steatosis [20,27].

### 4.2. Hepatokines as a Systemic Metabolic Communication Interface

Hepatokines are active mediators of inter-organ communication rather than mere metabolic biomarkers. By modulating insulin signaling, lipid pathways, and inflammatory responses, these biomolecules act as nutrient-sensitive molecular messengers [20,47]. Consequently, they convey the metabolic status of the liver to peripheral tissues, thereby aligning localized hepatic energy sensing with systemic metabolic regulation. Selected hepatokines, such as selenoprotein P and fetuin-A, promote insulin resistance by modulating signaling in muscle and adipose tissue and influencing endothelial function and cardiometabolic risk. These biomolecules are associated with maladaptive metabolic signaling.

In contrast, FGF21 is induced under conditions of metabolic stress and represents a key adaptive hepatokine. It promotes lipid catabolism and thermogenesis in brown adipose tissue, facilitating metabolic adaptation to energy overload and mitochondrial stress [13,20,30,47].

### 4.3. Liver–Peripheral Tissue Axis as an Energy Substrate Distribution System

Communication between the liver, adipose tissue, skeletal muscle, and pancreas is a fundamental mechanism of metabolic adaptation in pregnancy. This inter-organ network enables coordinated regulation of systemic energy allocation in response to changing metabolic demands.

Hepatokines induced by placental signals, such as selenoprotein P (SeP), inhibit glucose transport in muscle via suppression of protein kinase B (Akt) activity [18,19], thereby contributing to reduced peripheral glucose utilization and enhanced substrate availability for the fetoplacental unit.

In insulin-resistant states, compensatory β-cell proliferation occurs, partly regulated by hepatokines such as serpin B1, linking hepatic metabolic status to pancreatic adaptive capacity.

The liver–pancreas axis integrates β-cell secretory function with hepatocyte metabolic status [45]. Its disruption promotes insulin resistance and hepatic metabolic dysfunction, including steatosis with a pro-inflammatory hepatokine profile [15,20,48]. This underscores the bidirectional regulatory nature of liver–pancreas crosstalk in maintaining systemic metabolic homeostasis.

After delivery, this axis undergoes rapid reorganization due to declining placental hormones and changes in hepatokine profiles [18,37,43,49], reflecting a transition from a pregnancy-adapted metabolic state toward restoration of baseline hepatic–pancreatic signaling networks.

## 5. FGF21 in Pregnancy: Adaptive Metabolic Stress Signaling

### 5.1. The PPARα–FGF21 Axis as a Lipid Stress Sensor

The hepatic peroxisome proliferator-activated receptor alpha (PPARα) acts as a master lipid sensor and transcriptional regulator of genes involved in energy homeostasis and inflammatory responses [50,51]. It is activated in highly oxidative tissues, primarily the liver, where it integrates metabolic signals related to substrate availability. During pregnancy, this pathway contributes to maternal metabolic resilience by coordinating adaptive responses to progressive lipid mobilization and fluctuating energy demand.

PPARα activity depends on heterodimerization with retinoid X receptor (RXR), enabling regulation of genes involved in lipid and glucose metabolism, repair processes, and cell differentiation [50]. Its ligands include free fatty acids and endogenous molecules such as phospholipids and bilirubin. This ligand-dependent signaling network enables PPARα to function as an interface between nutrient sensing, endocrine adaptation, and hepatic metabolic programming during gestation.

This ligand—dependent activation positions PPARα as the primary upstream transcriptional driver required for subsequent FGF21 expression under conditions of elevated lipid flux. FGF21 may represent a downstream endocrine effector of PPARα signaling that potentially coordinates systemic metabolic adaptation to hepatic lipid stress [50,52].

### 5.2. FGF21 as a Metabolic Stress Hormone

These fluctuations reflect its role as a nutrient-sensitive regulator of substrate utilization.

FGF21 is a hepatokine secreted in response to energy deficit, fasting, and changes in substrate availability, acting as an endocrine regulator of appetite and energy fuel utilization [13,30,51]. During pregnancy, FGF21 is thought to function as a systemic stress-adaptation signal that coordinates maternal metabolic flexibility under conditions of physiological metabolic overload [12,13,30,53]. It improves insulin sensitivity and reduces lipotoxicity, thereby supporting metabolic adaptation to energy stress.

Through these mechanisms, FGF21 promotes metabolic flexibility by optimizing substrate utilization and limiting lipid-induced cellular stress. In chronic metabolic disease, elevated FGF21 likely reflects a compensatory response that is insufficient to restore metabolic homeostasis, consistent with the concept of FGF21 resistance [4,12,13,25,27,30,53,54,55].

### 5.3. FGF21 as a Response to Pregnancy-Induced Lipid Overload

In the third trimester, physiological hyperlipidemia and intense mobilization of fatty acids from adipose tissue occur, a process termed “lipid overflow” [30]. This state represents a physiological form of metabolic stress in which maternal tissues must adapt to sustained lipid exposure while maintaining metabolic homeostasis.

In this context, the liver–adipose axis must continuously balance lipid disposal with prevention of ectopic lipid accumulation.

FGF21 acts as a protective mechanism by limiting lipotoxicity through inhibition of de novo lipogenesis (including suppression of sterol regulatory element-binding protein 1c, SREBP-1c), thereby reducing hepatic metabolic burden [27]. This positions FGF21 as a hepatic stress-response factor that restrains lipid overaccumulation and preserves metabolic integrity under conditions of sustained lipid flux.

It also promotes browning of adipose tissue and increases thermogenesis, facilitating dissipation of excess energy as heat [12,13]. This mechanism creates a feedback loop between liver and adipose tissue, stabilizing circulating lipid levels and preventing transition from physiological hyperlipidemia to pathological states [13,30]. Accordingly, the FGF21 axis may represent a central component of interorgan communication linking hepatic lipid sensing with adipose tissue metabolic adaptation during pregnancy.

In conditions of excessive or prolonged metabolic stress, these adaptive mechanisms may become insufficient, potentially contributing to progression toward maladaptive metabolic states [12].

### 5.4. FGFR1/β-Klotho Signaling and FGF21 Resistance

Full FGF21 activity requires cooperation with the co-receptor β-Klotho and fibroblast growth factor receptors (FGFRs), which activate intracellular signaling cascades in target tissues [13,27,56]. FGFR1/β-Klotho activation induces kinase signaling and downstream transcriptional programs involved in lipid utilization and thermogenic regulation [13,14,56].

Reduced β-Klotho expression in adipose tissue has been proposed as an early marker of impaired FGF21 signaling, limiting thermogenic response [51]. In GDM, impaired receptor–cofactor availability may therefore contribute to reduced signaling efficiency and FGF21 resistance, despite elevated circulating ligand levels [13,14,27,51,56].

FGF21 activates adenosine monophosphate-activated protein kinase (AMPK), which increases GLUT4 translocation in skeletal muscle, enhancing insulin-independent glucose uptake [27]. This mechanism may help maintain maternal glucose homeostasis despite the physiological decline in insulin sensitivity observed during pregnancy.

AMPK simultaneously inhibits acetyl-CoA carboxylase (ACC) and activates carnitine palmitoyltransferase 1 (CPT1), promoting mitochondrial β-oxidation of fatty acids [27,57].

The FGF21–AMPK interaction forms a regulatory network controlling autophagy and mitophagy, reducing cellular stress and supporting metabolic adaptation under increased energy demand.

Adiponectin enhances this signaling axis, integrating communication between liver and adipose tissue [13,19,57,58].

### 5.5. Mitochondria as Effectors of FGF21 Action

FGF21 promotes mitochondrial biogenesis via activation of the sirtuin 1 (SIRT1)–peroxisome proliferator-activated receptor gamma coactivator 1-alpha (PGC-1α) axis, increasing mitochondrial number and ATP production efficiency [58].

It also reduces reactive oxygen species (ROS) production and improves electron transport chain efficiency, maintaining redox balance under metabolic stress.

FGF21 induces uncoupling proteins (UCP1–UCP3), enabling controlled energy dissipation as heat and reducing lipotoxicity [12,30]. This mechanism may prevent mitochondrial overload during physiological “lipid overflow” and facilitate safe disposal of excess energy substrates.

### 5.6. FGF21 as a Lipotoxicity Buffer and Regulator of Postpartum Adaptation

In late pregnancy, FGF21 exerts cytoprotective effects, protecting the liver, heart, and peripheral tissues from excess free fatty acids via activation of the SIRT1–AMPK axis [12,30,58].

It has anti-inflammatory and antioxidant properties, including induction of superoxide dismutase 2 (SOD2) and reduction in oxidative stress in cardiomyocytes [59].

After delivery, elevated FGF21 supports lipid mobilization and metabolic adaptation to lactation, although its long-term role in improving insulin sensitivity remains unclear [30,60].

In women with prior GDM, it may play a compensatory role; however, there is no conclusive evidence for its protective effect against the development of T2DM [60,61].

Whether persistent elevation of FGF21 after pregnancy reflects successful metabolic recovery or compensatory stress signaling remains unresolved.

## 6. SHBG Beyond Steroid Transport: Indicator and Mediator of Hepatic Insulin Sensitivity

### 6.1. Hepatic Regulation of SHBG

Sex steroid hormones, including androgens and estrogens, play important roles not only in reproductive function but also in energy metabolism [62]. They influence insulin secretion, β-cell function, insulin sensitivity, and glucose uptake [63]. According to the free hormone hypothesis, only unbound sex hormones are biologically active [64]. Since hormone activity depends on their bioavailability, SHBG plays a key role in regulating their effects [65].

SHBG is a glycoprotein produced mainly in the liver. Its expression is regulated by the transcription factor hepatocyte nuclear factor 4α (HNF4α) [66]. HNF4α is increasingly recognized not only as a regulator of SHBG transcription but also as an important determinant of hepatocyte metabolic identity, integrating glucose and lipid metabolism with endocrine signaling. Thus, HNF4α represents a central node linking hepatocyte transcriptional identity with endocrine output, including SHBG synthesis.

Several pathways linked to energy metabolism, inflammation, and lipid homeostasis, including AMPK, PPARγ, adiponectin, and pro-inflammatory cytokines, influence SHBG production. Also, conditions associated with insulin resistance, hepatic fat accumulation, obesity, and chronic low-grade inflammation are generally linked to lower circulating SHBG levels [67,68,69,70,71,72,73,74,75]. These observations support the concept that low SHBG reflects impaired hepatic metabolic regulation rather than an isolated hormonal abnormality.

Accordingly, SHBG should be interpreted as a functional surrogate marker of hepatic metabolic and transcriptional state rather than a passive transport protein.

### 6.2. SHBG Dynamics During Pregnancy and Postpartum

Pregnancy causes profound hormonal and metabolic changes that strongly affect SHBG levels. One of the main reasons for the increase in SHBG during pregnancy is the rising concentration of estrogens, which stimulate hepatic SHBG production [76]. SHBG levels increase early in pregnancy and continue to rise throughout gestation, reaching peak values in the third trimester. Higher SHBG concentrations help regulate the amount of free sex hormones in circulation and support hormonal balance during pregnancy [77].

The pregnancy-associated rise in SHBG represents a physiological paradox in which estrogen-driven stimulation of SHBG synthesis partially counterbalances the suppressive effects of insulin resistance on hepatic SHBG production. These endocrine adaptations contribute to the maintenance of pregnancy [78].

Abnormal sex steroid concentrations during the peripartum period may be associated with maternal complications, including preeclampsia, gestational diabetes, altered autoimmune disease activity, impaired cognition, structural brain changes, and increased breast cancer risk later in life [79].

Pregnancy is also characterized by progressive insulin resistance, particularly in the second and third trimesters. Although insulin resistance generally suppresses SHBG production, the strong stimulatory effect of estrogens predominates during pregnancy, resulting in persistently elevated SHBG levels [77].

Nevertheless, women with obesity, GDM, or excessive gestational weight gain often have lower SHBG levels than healthy pregnant women [80]. This suggests that metabolic disturbances may attenuate the physiological pregnancy-related rise in SHBG. In pregnancies complicated by GDM or pregestational obesity, insulin-mediated suppression of hepatic SHBG synthesis may become more apparent despite sustained estrogenic stimulation.

After delivery, placental separation leads to a rapid decline in hormone concentrations and substantial endocrine changes in the postpartum period. Estrogen and progesterone levels decrease, and SHBG concentrations gradually return toward pre-pregnancy values over subsequent weeks and months [77]. This decline reflects both reduced estrogen stimulation and postpartum metabolic adaptations [79].

Additionally, breastfeeding is associated with improved glucose metabolism, enhanced insulin sensitivity, and mobilization of maternal fat stores [81,82]. These changes may contribute to a more favorable metabolic profile and potentially influence SHBG regulation.

### 6.3. SHBG as a Surrogate of Hepatic Insulin Responsiveness

SHBG is increasingly recognized as a marker of metabolic health and hepatic insulin sensitivity. Because SHBG is produced mainly in hepatocytes, circulating SHBG levels reflect changes in liver metabolism and insulin signaling. Clinical and experimental studies have shown that low SHBG concentrations are associated with insulin resistance, obesity, T2DM, metabolic dysfunction-associated steatotic liver disease (MASLD), and polycystic ovary syndrome (PCOS) [69,75,83,84,85]. These findings suggest that SHBG may represent a marker of hepatic metabolic dysfunction rather than merely a transport protein for sex hormones.

Accumulating evidence indicates that the relationship between insulin resistance and SHBG is strongly linked to hepatic lipid metabolism. In vitro and animal studies demonstrated that monosaccharides such as glucose and fructose are converted into palmitate, which suppresses HNF4α and subsequently decreases SHBG synthesis. This suggests that low SHBG concentrations may reflect hepatic lipid overload and impaired hepatic insulin responsiveness rather than the isolated effect of hyperinsulinemia [84].

Current evidence also supports the concept of selective hepatic insulin resistance, in which insulin loses its ability to suppress hepatic gluconeogenesis while lipogenic pathways remain active or become overstimulated [86,87]. As a result, hepatic triglyceride synthesis and liver fat accumulation continue despite systemic insulin resistance [88]. Persistent activation of lipogenic pathways may therefore contribute to reduced SHBG production.

Lower SHBG levels are associated not only with hepatic fat accumulation but also with markers of liver injury and impaired metabolic homeostasis [85]. Inflammation and adipose tissue dysfunction further contribute to SHBG regulation. Obesity and insulin resistance are associated with chronic low-grade inflammation, elevated TNF-α and IL-1β concentrations, and reduced adiponectin levels [89,90]. These mediators influence HNF4α expression and suppress SHBG synthesis. Adiponectin appears to support HNF4α activity through AMPK, whereas inflammatory cytokines inhibit HNF4α through NF-κB and MAPK signaling pathways [67]. Consequently, reduced SHBG concentrations may integrate several components of metabolic dysfunction, including hepatic insulin resistance, inflammation, adipose tissue dysfunction, and lipid accumulation.

Genome-wide association studies (GWAS) identified several loci associated with circulating SHBG levels in genes involved in hepatic glucose and lipid metabolism, including GCKR, GCK, MLXIPL/ChREBP, HNF4A, and PNPLA3 [91]. One of the strongest associations was identified for the GCKR variant rs1260326, which encodes glucokinase regulatory protein (GKRP), a regulator of hepatic glucokinase activity. Studies showed that this variant reduces the inhibitory effect of GKRP on glucokinase, leading to increased hepatic glucose uptake, enhanced glycolysis, and increased de novo lipogenesis with subsequent liver fat accumulation [92,93].

Lower SHBG levels were also associated with variants in GCK, encoding glucokinase, and MLXIPL, encoding carbohydrate response element-binding protein (ChREBP), a key transcription factor regulating lipogenic enzymes [94].

From a systems biology perspective, SHBG may be viewed as an integrative marker of hepatic insulin signaling and metabolic status, reflecting the combined influence of lipogenesis, inflammation, and broader hepatic transcriptional regulation. However, SHBG should be interpreted primarily as a marker of these processes rather than a direct mediator of metabolic adaptation. Nevertheless, whether SHBG itself contributes to these abnormalities or simply mirrors underlying metabolic disturbances remains uncertain. Taken together, these findings suggest that circulating SHBG levels are linked to pathways regulating hepatic glucose metabolism, lipogenesis, and intrahepatic lipid accumulation. Low SHBG concentrations appear to reflect impaired hepatic insulin sensitivity, increased de novo lipogenesis, liver fat accumulation, chronic inflammation, and adipose tissue dysfunction.

### 6.4. Postpartum Recovery of SHBG Regulation and Hepatic Metabolic Function

After delivery, hormone levels drop rapidly, and many pregnancy-related metabolic changes begin to reverse. Insulin sensitivity improves, liver fat production decreases, and glucose and lipid metabolism gradually move back toward the pre-pregnancy state [95,96].

Despite this, little is known about how SHBG changes during the postpartum period. Most studies have focused on gestational diabetes mellitus (GDM), postpartum glucose intolerance, or future diabetes risk [67,83,97,98]. Studies specifically examining SHBG after pregnancy are still limited.

Recovery of normal hepatic metabolic regulation may contribute to restoration of SHBG synthesis during the postpartum period, although the underlying mechanisms remain incompletely characterized. Accordingly, SHBG should be considered not only as a reproductive hormone-binding protein but also as a hepatic marker linked to metabolic regulation [99].

Several studies demonstrated associations between SHBG levels, hyperinsulinemia, and GDM [100,101]. Women with previous GDM also remain at increased risk of postpartum glucose intolerance and later T2DM [102], suggesting that metabolic recovery after pregnancy may be incomplete in some patients.

Low SHBG levels after delivery could indicate persistent insulin resistance or delayed metabolic recovery, and are consistently associated with poor metabolic health, although there is currently no clear evidence that SHBG itself drives these changes.

Another point worth considering is the source of circulating SHBG during pregnancy. Although SHBG is produced mainly by the liver, expression has also been reported in placental tissue. As a result, circulating SHBG levels during pregnancy may not reflect liver function alone. The relative contribution of hepatic and placental SHBG remains unclear.

## 7. Integrated Molecular Model: Coordinated Hepatokine Control of Postpartum Insulin Sensitivity

### 7.1. Converging Molecular Pathways Regulating Metabolic Plasticity

Pregnancy constitutes a dynamic metabolic state characterized by progressive insulin resistance, enhanced lipolysis, and increased hepatic glucose output. Although these adaptations are physiologically required to ensure adequate nutrient supply to the fetus, they impose significant metabolic stress on maternal tissues [32]. Restoration of insulin sensitivity after delivery is not solely driven by the withdrawal of placental hormones, but rather reflects hepatic metabolic reprogramming involving hepatokine signaling [103,104].

Within this framework, the FGF21-associated axis is interpreted as reflecting metabolic stress response capacity, whereas SHBG-associated variation is considered a proxy of hepatic insulin sensitivity and transcriptional state [105,106]. A summary of the integrated signaling nodes is provided in Table 1.

Several intracellular nodes integrate these axes:

- AMPK, acting as a cellular energy sensor, is activated under metabolic stress and promotes fatty acid oxidation, mitochondrial biogenesis, and improved insulin signaling. FGF21 has been shown to enhance AMPK activity in peripheral tissues, thereby supporting adaptive fuel utilization during periods of increased lipid flux [108];
- PPARα, a key transcriptional regulator of hepatic β-oxidation, drives FGF21 expression in response to lipid overflow and fasting-like signals characteristic of late pregnancy. Activation of the PPARα–FGF21 pathway may therefore buffer lipotoxic stress and limit ectopic lipid accumulation [109];

These pathways primarily mediate acute energetic adaptation and lipid handling.

- PI3K–Akt signaling, central to insulin action, is progressively attenuated during gestational insulin resistance. Restoration of this pathway postpartum likely reflects recovery of hepatic insulin responsiveness. SHBG production, which is suppressed by hyperinsulinemia, may serve as a functional indicator of hepatic insulin sensitivity and HNF4α-dependent transcriptional activity, thereby reflecting hepatic insulin action [110];
- HNF4α, a master regulator of hepatocyte metabolic identity, controls SHBG transcription and coordinates genes involved in glucose and lipid metabolism. Impaired HNF4α activity under conditions of insulin resistance and hepatic steatosis may contribute to reduced SHBG levels, whereas postpartum normalization of hepatic nutrient handling and endocrine signaling may restore HNF4α-driven transcriptional programs [73].

Together, these interconnected pathways suggest that FGF21 and SHBG operate within a shared regulatory network governing hepatic adaptive plasticity, with complementary roles in acute metabolic buffering and long-term transcriptional reprogramming.

### 7.2. A Dynamic Three-Phase Model of Hepatokine-Mediated Adaptation

We propose a dynamic, phase-dependent model of hepatokine regulation across pregnancy and the postpartum period.

#### 7.2.1. Early Pregnancy: Relative Insulin Sensitivity and Metabolic Priming

In early gestation, maternal insulin sensitivity is relatively preserved or even transiently enhanced. Hepatic energetic and transcriptional programs remain largely balanced, and SHBG levels may reflect intact HNF4α activity and insulin signaling [73]. FGF21 activity is moderate, consistent with limited metabolic stress.

#### 7.2.2. Late Pregnancy: Adaptive Insulin Resistance and Metabolic Resilience

As gestation progresses, placental hormones promote systemic insulin resistance, increased adipose tissue lipolysis, and elevated circulating free fatty acids. This lipid overflow challenges hepatic metabolic capacity [111].

In this context, activation of the PPARα–FGF21 axis may function as a compensatory mechanism that

- Enhances fatty acid oxidation;
- Supports ketogenesis when required;
- Limits hepatic lipotoxicity;
- Maintains mitochondrial function.

Thus, FGF21 may act as a resilience factor that permits physiological insulin resistance without irreversible metabolic damage [112,113].

Concurrently, hyperinsulinemia and altered hepatic lipid flux may be associated with reduced SHBG production, potentially reflecting impaired hepatic insulin sensitivity via HNF4α-dependent transcription.

#### 7.2.3. Postpartum Period: Active Metabolic Resetting

Following delivery, the rapid decline in placental hormones reduces diabetogenic pressure, but full restoration of insulin sensitivity requires coordinated hepatic reprogramming rather than passive hormonal normalization alone [15]. Postpartum recovery may involve:

1. Attenuation of lipid overflow and inflammatory signaling;
2. Persistence or recalibration of FGF21-mediated metabolic adaptations, facilitating the transition from a lipolytic to a balanced metabolic state;
3. Reactivation of HNF4α-dependent transcription and restoration of SHBG production, associated with improved hepatic insulin responsiveness and re-engagement of PI3K–Akt signaling [114,115].

**Figure 1:**
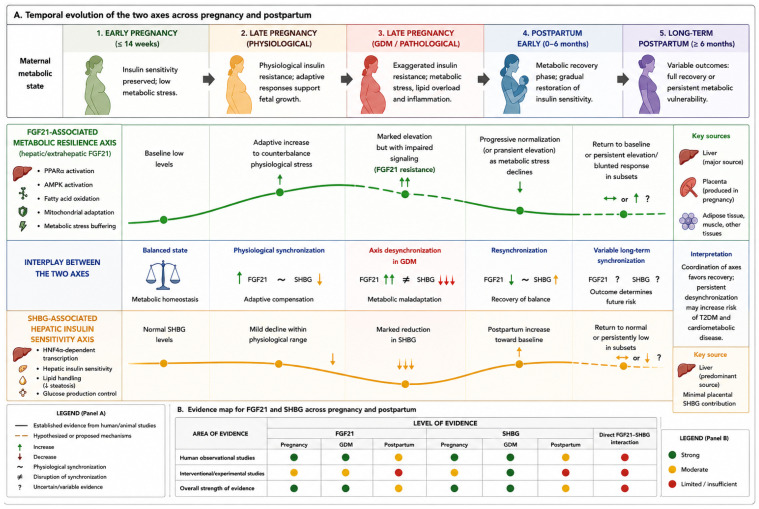
Proposed trajectories of FGF21 and SHBG across pregnancy and postpartum. (**A**) Conceptual model illustrating temporal changes in the FGF21-associated metabolic resilience axis and the SHBG-associated hepatic insulin sensitivity axis during physiological pregnancy, GDM, and postpartum recovery. Solid lines indicate evidence-supported observations, whereas dashed lines denote hypothesized or insufficiently validated mechanisms. (**B**) Evidence map summarizing the strength of current evidence for FGF21 and SHBG across pregnancy, GDM, and the postpartum period, and for their potential interaction.

Postpartum recovery is therefore conceptualized as a two-layer process: metabolic recalibration followed by transcriptional identity restoration (Figure 1).

### 7.3. Pathophysiological Implications: When Synchronization Fails

A proposed feature of this model is a potential temporal association between FGF21-related metabolic adaptations and changes in SHBG levels [116].

It has been hypothesized that disruption in the regulation of FGF21 and SHBG pathways may be associated with persistent insulin resistance after GDM. However, direct evidence supporting coordinated regulation between these pathways is lacking. For example:

- Altered FGF21 signaling has been proposed in association with impaired metabolic adaptation during late pregnancy, although causal links to hepatic steatosis and persistent dysfunction remain unproven.
- Changes in circulating SHBG after pregnancy have been associated with hepatic insulin sensitivity; however, whether this reflects delayed normalization of specific transcriptional regulation remains unclear.
- Inflammation and ectopic lipid accumulation have been implicated in metabolic dysfunction after GDM and may influence multiple hepatic signaling pathways [13,117].

Women with prior GDM remain at increased risk of T2DM and cardiometabolic disease, although the mechanistic basis remains incompletely understood [118]. Postpartum metabolic alterations likely reflect a multifactorial process involving incomplete resolution of pregnancy-associated metabolic and hormonal changes.

### 7.4. Conceptual Implications

This integrated hepatokine model proposes pregnancy as a physiological experiment in reversible insulin resistance, in which the liver may play an endocrine role in potentially influencing whether metabolic stress resolves or progresses toward chronic disease [95].

Rather than viewing postpartum insulin sensitivity as a passive rebound phenomenon, our framework proposes that coordinated hepatokine signaling—through the metabolic resilience axis (FGF21) and the hepatic SHBG-associated insulin sensitivity—may contribute to variability in metabolic recovery after pregnancy.

Overall, pregnancy and the postpartum transition may be conceptualized as a continuum of regulated hepatic endocrine plasticity, rather than two discrete metabolic states.

From a translational perspective, this framework may help guide the future development of postpartum risk-stratification strategies integrating hepatokine profiling, hepatic insulin-sensitivity markers, and longitudinal metabolic surveillance following GDM. In particular, the combined assessment of FGF21 and SHBG trajectories may assist in identifying women at increased risk of persistent insulin resistance and future T2DM progression (Table 2).

At present, longitudinal and mechanistic studies are required to determine whether these pathways merely reflect or actively influence postpartum metabolic trajectories.

## 8. Clinical Implications of the Proposed Two-Axis Hepatokine Framework

### 8.1. Integrative Framing

FGF21 and SHBG reflect different aspects of liver metabolism. In this review, FGF21 is discussed as a marker of the response to metabolic stress, whereas SHBG is viewed as a marker of hepatic insulin sensitivity and metabolic health [119,122]. Based on these observations, we propose a model in which both markers provide information about metabolic adaptation during pregnancy. However, there is currently no direct evidence that FGF21 and SHBG are linked through a common regulatory pathway. This framework should be considered hypothesis-generating rather than mechanistically established.

### 8.2. Prediction of GDM and Early Metabolic Risk Stratification

The proposed two-axis framework (FGF21-associated metabolic resilience axis and SHBG-associated hepatic insulin sensitivity axis) provides a conceptual extension of traditional static glycemic markers for early risk stratification in pregnancy.

In this context, circulating FGF21 may reflect metabolic stress associated with lipid overload and mitochondrial dysfunction, whereas SHBG levels may serve as a surrogate marker of hepatic insulin sensitivity and HNF4α-associated transcriptional activity [75,123,124].

Combined assessment of these hepatokine-associated pathways may hypothetically improve early identification of women transitioning from physiological insulin resistance to pathological gestational glucose dysregulation [98]. However, findings across studies have not always been consistent. In addition, it remains unclear whether the measurement of both biomarkers improves risk prediction beyond established clinical and biochemical risk factors. Further prospective studies are needed before their clinical value can be determined.

### 8.3. Prediction of Long-Term Risk of Type 2 Diabetes After Pregnancy

Pregnancy complicated by GDM represents a period of transient metabolic stress during which underlying hepatic insulin resistance may become clinically apparent. Women with previous GDM are at increased risk of developing type 2 diabetes and other cardiometabolic disorders later in life.

Persistent alterations in FGF21 and SHBG after delivery may reflect ongoing metabolic dysfunction and could potentially identify women at higher long-term risk [120,121,122,123,124,125,126].

At present, FGF21 and SHBG should be regarded as promising research biomarkers rather than validated predictors of long-term metabolic outcomes.

### 8.4. Therapeutic Hypotheses Targeting the FGF21-Associated Axis

Several interventions that improve metabolic health may also influence FGF21 signaling. These include lifestyle modification, dietary interventions, physical activity, and emerging FGF21-based therapies [127,128]. From a mechanistic perspective, such interventions may enhance FGF21–PPARα–AMPK signaling, thereby improving lipid oxidation, mitochondrial function, and resistance to lipotoxic stress.

However, the interpretation of circulating FGF21 levels is complicated by the phenomenon of FGF21 resistance. Elevated FGF21 concentrations may reflect compensatory responses to metabolic stress rather than preserved biological activity. This should be considered when evaluating the clinical relevance of FGF21 measurements.

### 8.5. Modulation of Hepatic Insulin Sensitivity and the SHBG-Associated Axis

Improvement in hepatic insulin sensitivity may represent a potential mechanism for normalization of SHBG levels during and after pregnancy.

Possible pathways include reduction in hepatic lipogenesis, attenuation of inflammatory signaling, and restoration of insulin-regulated hepatic transcriptional programs, potentially involving HNF4α-associated networks [107]. Collectively, these processes converge on improved hepatocyte metabolic function and reduced lipogenic drive, which may permit reactivation of SHBG transcriptional output.

Given its clinical accessibility, SHBG may serve as a pragmatic biomarker for monitoring hepatic insulin sensitivity dynamics during postpartum metabolic recovery. However, its role as a direct surrogate of hepatic transcriptional integrity remains indirect and not yet mechanistically validated in longitudinal human studies.

In this context, SHBG should be interpreted primarily as a circulating biomarker reflecting hepatic insulin sensitivity and HNF4α-dependent transcriptional activity rather than an active mediator of insulin signaling. Accordingly, changes in SHBG levels are best understood as downstream consequences of hepatic metabolic reprogramming rather than drivers of insulin action.

### 8.6. Combined Preventive and Therapeutic Strategy

Integrated modulation of both proposed axes may offer a more comprehensive approach to metabolic risk reduction than targeting either pathway alone.

Within this conceptual framework, simultaneous assessment of FGF21 and SHBG trajectories is more appropriately interpreted as reflecting coordinated changes in acute metabolic stress responses and longer-term hepatic metabolic reprogramming.

This model conceptualizes pregnancy as a dynamic period of hepatic endocrine adaptation with potential long-term cardiometabolic consequences extending beyond glycemic regulation alone.

However, the clinical utility of integrated FGF21-SHBG profiling remains to be established. At present, this framework should be interpreted as a systems-level hypothesis requiring validation in longitudinal clinical and mechanistic studies. In particular, its immediate translational value is currently limited to risk stratification and hypothesis generation rather than interventional guidance.

## 9. Conclusions

Pregnancy represents a unique physiological state of coordinated metabolic stress and adaptation, characterized by a reversible shift toward systemic insulin resistance that ensures adequate nutrient allocation to the developing fetus. Rather than being a purely passive consequence of placental hormone action, this state appears to arise from an integrated network of hepatic signaling pathways that may contribute to maternal metabolic flexibility.

In this framework, the liver may act as a key endocrine organ involved in systemic metabolic regulation through hepatokine-mediated communication. Two complementary regulatory axes can be distinguished: a rapid metabolic resilience axis centered on FGF21, and a slower hepatic insulin-sensitivity-related axis reflected by SHBG levels. Together, these pathways integrate lipid flux, glucose homeostasis, inflammatory tone, and transcriptional control of hepatic function.

FGF21 is considered an adaptive stress-response hormone that may contribute to the regulation of lipid metabolism, fatty acid oxidation, and mitochondrial function through AMPK- and PPARα-related pathways. While FGF21 may reflect dynamic metabolic stress responses, SHBG may provide a more stable circulating biomarker of hepatic insulin-related metabolic status.

The potential interaction between these axes may offer a conceptual framework of gestational insulin resistance and postpartum metabolic recovery. During pregnancy, physiological insulin resistance is maintained within adaptive limits through FGF21-mediated metabolic buffering, while SHBG dynamics reflect changes in hepatic insulin signaling. After delivery, restoration of metabolic homeostasis requires not only withdrawal of placental hormones but also resolution of pregnancy-related endocrine influences and gradual re-establishment of hepatic metabolic regulation.

Disruption of this coordinated system may contribute to pathological outcomes, including GDM and increased long-term risk of T2DM. In such cases, reduced metabolic flexibility, persistent hepatic insulin resistance, or incomplete normalization of hepatic transcriptional regulation may prevent full postpartum metabolic recovery.

Overall, the proposed two-axis hepatokine model conceptualizes pregnancy and the postpartum period as a continuum of hepatic endocrine adaptation. In this context, insulin resistance may represent a physiological response with potential for dysregulation. Future longitudinal and mechanistic studies are required to clarify whether FGF21 and SHBG act as circulating biomarkers or play active roles in maternal metabolic regulation.

## Figures and Tables

**Table 1:** Dynamic interpretation of FGF21 and SHBG across pregnancy and postpartum.

Stage	FGF21 (Metabolic Axis)	SHBG (Hepatic Identity Axis)	Interpretation	References
Early pregnancy	Low–moderate	Normal	Baseline hepatic metabolic balance	[30,77,78]
Late pregnancy (physiological IR)	High (adaptive stress response)	Moderately reduced or stable (estrogen offset)	Compensated insulin resistance	[14,30,80,84,107]
Late pregnancy (GDM risk state)	High but dysregulated/resistant state	Low (reflecting reduced hepatic insulin sensitivity)	Impaired hepatic metabolic adaptation + lipotoxic stress	[30,83,84,97,98,101]
Postpartum recovery	Gradual normalization or transient elevation	Rising toward baseline	Hepatic reprogramming and insulin sensitivity restoration	[30,98]
Post-GDM state	Persistently elevated or blunted	Persistently low (reflecting sustained hepatic insulin resistance)	Impaired axis resynchronization → increased T2DM risk	[14,15,84,98,101,103]

**Table 2:** Strengths, limitations, and knowledge gaps of the proposed FGF21–SHBG dual-axis model of maternal metabolic adaptation.

Proposed Component	Supporting Evidence	Limitations	Research Priorities	References
FGF21 rises during pregnancy as an adaptive metabolic response	Multiple human observational studies; experimental studies supporting a role in lipid oxidation and metabolic stress adaptation	Considerable variability between studies; contribution of placental FGF21 remains uncertain	Standardized longitudinal studies across pregnancy	[30,54,61,119,120]
Elevated FGF21 is associated with GDM	Consistent findings in several cohorts	Direction of causality remains unclear; evidence of FGF21 resistance complicates interpretation	Mechanistic studies evaluating FGF21 signaling competence in GDM	[14,30,60]
SHBG is inversely associated with insulin resistance and GDM risk	Strong epidemiological evidence; biological plausibility through HNF4α regulation	SHBG may function primarily as a biomarker rather than a direct mediator	Studies differentiating biomarker versus causal effects	[83,84,97,101]
Postpartum recovery is accompanied by normalization of FGF21 and SHBG levels	Limited observational evidence	Few longitudinal postpartum studies; heterogeneous follow-up periods	Long-term postpartum metabolic phenotyping	[107,118,121]
Coordinated regulation of FGF21 and SHBG reflects restoration of hepatic metabolic homeostasis	Indirect evidence from shared associations with insulin sensitivity and hepatic metabolism	No direct evidence demonstrating coordinated regulation	Simultaneous longitudinal assessment of FGF21, SHBG, and hepatic metabolic markers	[13,14,20,30,47,84,119]
Direct crosstalk between FGF21 and SHBG pathways	Biological plausibility through shared upstream metabolic regulators	No direct experimental or clinical evidence	Mechanistic studies investigating interactions between the two pathways	[13,14,67,99,110]

## Data Availability

No new data were created or analyzed in this study. Data sharing is not applicable to this article.
